# Fabrication of Catalytic Distillation Membranes with Atomic Layer Deposition

**DOI:** 10.1021/acsami.6c07890

**Published:** 2026-07-03

**Authors:** Elizabeth A. Hjelvik, Kian P. Lopez, Samuel D. Marks, Bryce Knutson, Scott C. Minas, Madison King, Joseph C. Roback, Matthew Ticknor, Ryan C. Hayward, Michael F. Toney, John Watt, Dale L. Huber, Anthony P. Straub

**Affiliations:** † Center for Integrated Nanotechnologies, 1105 Sandia National Laboratories, Albuquerque, New Mexico 87123, United States; ‡ Department of Materials Science and Engineering, 1877 University of Colorado Boulder, Boulder, Colorado 80309, United States; § Department of Chemical and Biological Engineering, University of Colorado Boulder, Boulder, Colorado 80309, United States; ∥ Nanoscience and Microsystems Engineering, 1104 University of New Mexico, 210 University Blvd NE, Albuquerque, New Mexico 87131, United States; ⊥ Advanced Photon Source, Argonne National Laboratory, Lemont, Illinois 60439, United States; # Center for Integrated Nanotechnologies, 5112 Los Alamos National Laboratory, Los Alamos, New Mexico 87545, United States; ∇ 3356 Northern Arizona University, Center for Materials Interfaces in Research and Applications, Flagstaff, Arizona 86011, United States; ○ Renewable and Sustainable Energy Institute, University of Colorado Boulder, Boulder, Colorado 80309, United States; ◆ Department of Mechanical and Process Engineering, ETH Zürich, Zürich 8092, Switzerland

**Keywords:** distillation, photocatalytic, atomic layer deposition, membrane processes, desalination

## Abstract

The integration of catalysts onto the surface of membranes enables simultaneous physical separation and catalytic transformation of constituents in a feed stream, facilitating improved contaminant removal and fouling mitigation. Distillation membranes are a particularly attractive platform for catalytic membranes because they reject nonvolatile species and exhibit exceptional resistance to oxidative and radical-driven degradation. However, imparting catalytic functionality onto hydrophobic, porous distillation membranes has proven challenging since the membranes used are chemically inert and difficult to modify. Furthermore, catalysts on the membrane surface can decrease hydrophobicity and increase the membrane’s susceptibility to pore wetting and failure. In this work, we create a catalytic distillation membrane by coating a polytetrafluoroethylene membrane surface with titanium dioxide (TiO_2_) via plasma-assisted atomic layer deposition (ALD). By precisely tuning the ALD parameters, we demonstrate localized growth of TiO_2_ near (within approximately 1 μm) the surface of polytetrafluoroethylene membranes, forming a catalytically active interface while preserving the underlying hydrophobic pore structure. Localized growth of TiO_2_ is confirmed by electron microscopy and spectroscopy techniques, and membranes coated with 500 cycles of ALD show pressure tolerance up to 12.8 bar and higher than 95% salt rejection in pressure-driven distillation. Photocatalytic activity is demonstrated via the degradation of methylene blue dye under UV irradiation, where increasing TiO_2_ loading leads to an enhancement in dye degradation. These results establish a general strategy for integrating catalytic functionality into chemically inert, hydrophobic membranes without compromising distillation performance, providing a pathway toward multifunctional membranes that couple advanced oxidation with membrane separation for water treatment.

## Graphical abstract

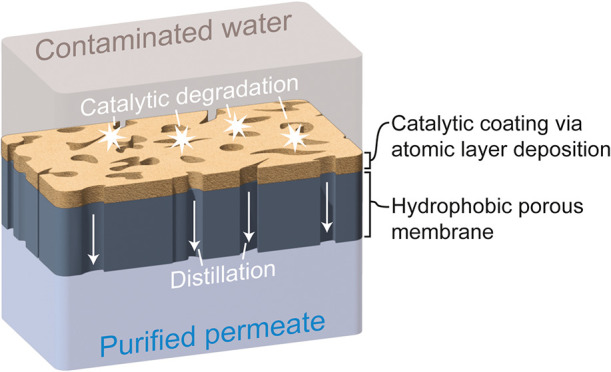

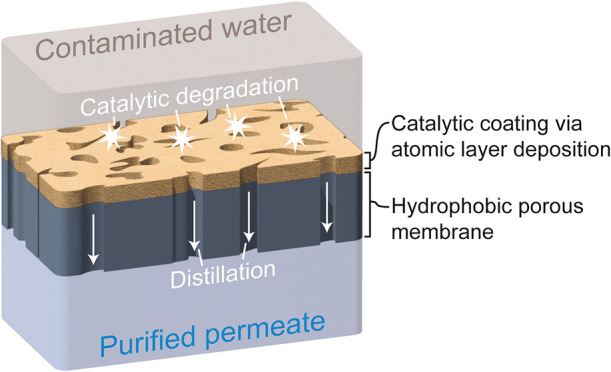

## Introduction

Increasing global water scarcity is driving the treatment of more complex and contaminated water sources, such as municipal and industrial wastewaters.^1–3^ In these waters, trace organic contaminants have emerged as a major challenge for advanced treatment systems due to their persistence, negative health effects, and resistance to conventional treatment processes.^3–5^ At the same time, the high organic content of many impaired waters increases fouling propensity in membrane-based advanced treatment processes, leading to performance losses, shortened membrane lifetimes, and increased operational costs.^6–8^ These challenges motivate the development of treatment technologies that can simultaneously achieve high selectivity, robust operation, and in situ degradation of problematic organic compounds.

Catalytic membrane processes have attracted growing interest as a strategy for coupling physical separation with chemical transformation. In these systems, membrane filtration, such as nanofiltration or reverse osmosis, is combined with catalytic reactions at the membrane surface to generate reactive species, such as hydroxyl radicals, sulfate radicals, or other oxidants, that can degrade organic contaminants.^9–11^ Such hybrid approaches offer the potential to reduce chemical dosing requirements, mitigate fouling through oxidative cleaning, and enable compact treatment architectures; however, existing catalytic membrane platforms face limitations. Reactive oxygen species generated during advanced oxidation can chemically degrade polymeric membrane materials, particularly polyamide active layers used in nanofiltration and reverse osmosis.^12–14^ In addition, catalytic coatings and embedded catalytic nanoparticles frequently reduce effective membrane permeability by blocking transport pathways or increasing hydraulic resistance.^15,16^ Finally, oxidation of larger organic molecules often produces low-molecular-weight byproducts that readily pass through conventional membranes, compromising water quality in the membrane permeate and raising concerns about transformation-product release.^17,18^

Distillation-based membranes provide a compelling alternative platform for catalytic membranes. Distillation membranes rely on vapor-phase transport across hydrophobic porous media, enabling inherently high rejection of nonvolatile species, including low-molecular-weight byproducts of catalytic oxidation.^19–21^ Distillation membranes are also commonly fabricated from chemically robust polymers such as polytetrafluoroethylene (PTFE), which exhibit exceptional resistance to oxidative and radical-driven degradation compared to polyamide-based membranes.^19^ Because transport occurs through gas-filled pores rather than dense polymers, distillation membranes can tolerate partial surface modification without obstructing permeation pathways.^20,22,23^ Together, these properties suggest that distillation membranes offer a unique opportunity to create catalytic membranes that are highly selective and robust.

Despite the promise of integrating catalytic functionality with distillation membranes, this technology has remained largely unexplored. A key barrier is the intrinsic chemical inertness of hydrophobic polymers used in distillation, which complicates catalyst attachment.^24–28^ Moreover, many catalytic materials, such as metal oxides, are hydrophilic and can compromise membrane hydrophobicity, leading to pore wetting and membrane failure.^29–31^ Existing coating approaches often coat the entire membrane, rendering the pores hydrophilic and accelerating membrane failure via pore wetting. Thus, strategies that limit the infiltration of chemical precursors into the membrane pores are needed to selectively modify only the membrane surface while preserving the hydrophobic porous matrix.^32–35^ Overcoming these fabrication challenges is essential for realizing the catalytic distillation membranes.

In this work, we demonstrate catalytically active distillation membranes based on plasma-assisted atomic layer deposition (ALD) of TiO_2_ onto commercial PTFE membranes. Using short precursor exposure times and surface-activated growth, we achieve TiO_2_ coatings with controlled penetration depth that preserve membrane porosity and vapor transport pathways. With a combination of electron microscopy, spectroscopy, and wettability characterization, we quantify coating thickness, chemical composition, and interfacial properties. We further demonstrate that TiO_2_-coated membranes maintain wetting resistance and desalination performance in pressure-driven distillation (PD) and exhibit catalytic activity, establishing a new method for integrating advanced oxidation with membrane separation. This approach provides a generalizable route toward chemically robust, multifunctional membranes capable of addressing emerging challenges in advanced water treatment.

## Materials and Methods

### Membranes and Chemicals

Commercial polytetrafluoroethylene (PTFE) membranes (model PTF002LH0P, nominal pore size 20 nm) were obtained from Cytiva. The membranes were hydrophobic and supported on a nonwoven backing layer, as provided by the manufacturer. For ALD reactions, trimethylaluminum (Sigma-Aldrich, USA), titanium tetrachloride (Sigma-Aldrich, USA), and deionized (DI) water were used as reaction precursors. Sodium chloride (Fisher Chemical, USA) dissolved in DI water was used as the feed solution in liquid entry pressure tests. Ultrapure water (resistivity ≥ 18.2 MΩ·cm) produced using a Milli-Q purification system was used for all experimental preparations and cleaning steps. Methylene blue (Sigma-Aldrich, USA) was dissolved in DI water and diluted to 5 mg/L for catalytic degradation tests.

### Atomic Layer Deposition

Plasma-assisted ALD was performed at 165 °C in a Picosun Sunale R150 ALD. Two reactions were programmed for deposition onto the PTFE surface. The first was a remote oxygen plasma step followed by the introduction of trimethylaluminum (TMA). For the plasma-TMA cycle, five cycles of remote oxygen plasma with a power of 2000 W, a pulse time of 30 s, and a purge time of 10 s were performed, and then five cycles of TMA with a pulse time of 0.1 s and a purge time of 6 s were performed. The second reaction was the TiO_2_ formation with titanium tetrachloride and water. TiCl_4_ was dosed into the ALD chamber with a pulse time of 0.1 s and a purge time of 6.0 s. Then, water was dosed with a pulse of 0.2 s and a purge of 8.0 s.

### Membrane Characterization

The membrane morphology and surface topography were viewed with scanning electron microscopy (SEM) on a Hitachi SU8010 microscope. Prior to SEM imaging, platinum was sputtered onto the PTFE membranes until a thickness of 3 nm was achieved.

Cross-sectioning and imaging of the PTFE membranes was performed on a Thermo Fisher Scientific Scios 2 focused ion beam/scanning electron microscope (FIB/SEM) with a Leica liquid nitrogen-cooled SEM stage. A 7 nm gold thin film was sputtered over the entire sample on an SEC MCM-100P ion sputter coater under vacuum to reduce surface charging under the electron and Ga-ion beam. The samples were cooled on the cryo-stage under the high-vacuum environment of the SEM to −150 °C for FIB cross-sectioning. Organometallic Pt precursor was deposited in the FIB and cured using the Ga-ion beam (30 kV, 1.0 nA) to yield a 1.5 μm thick protective Pt layer on the surface of the PTFE membranes. Ga-ion beam voltage was kept at 30 kV, but currents were kept below 5 nA for cuts and were polished with a current of 1.0 nA to prevent damage to the polymeric matrix. All energy-dispersive X-ray spectroscopy (XEDS) spectra were drift corrected and taken with an E-beam voltage and current of 10 kV and 0.2 nA, respectively, with a pixel dwell time of 1 ms. XEDS maps were taken using an EDAX Octane Elite detector.

XPS survey spectra (0–1200 eV) were acquired on a Kratos Supra spectrometer using an Al Kα source at 1486.7 eV and 15 mA emission current; charge neutralization parameters were determined via continuous C 1s scanning. High-resolution elemental core scans were collected on a Kratos Axis Ultra DLD spectrometer with a monochromatic Al Kα source at 120 W (1486.6 eV), a base pressure of 3 × 10^–9^ Torr, and a pass energy of 20 eV; charge compensation was achieved using low-energy electrons, and all spectra were referenced by setting the C 1s peak to 284.6 eV. Polymer samples were isolated on the sample plate to ensure consistent charging, and data were processed using CasaXPS. Survey spectra were used to determine elemental atomic percentages. Measurements were taken at four locations per sample across three replicates, and error bars reflect the standard deviation of these measurements.

Contact angles were measured with a Biolin Scientific tensiometer using the sessile drop technique. For all measurements, a droplet of DI water (less than 10 μL) was deposited onto the active side of the membrane surface using a pipette.

Grazing-incidence wide-angle X-ray diffraction profiles were acquired using Cu Kα radiation (Xenocs Xeuss 3.0) on the 1000TiO_2_ sample. Samples were mounted onto a silica substrate. The X-ray angle of incidence was 0.2° to maximize sensitivity to the membrane surface structure.

The presence and morphology of TiO_2_ on the membrane surface were investigated using X-ray computed tomography (XCT) imaging in the absorption contrast imaging modality of a Zeiss Xradia Ultra 810 lab instrument with large field of view (LFOV) optics. The PTFE membrane was prepared with a cold fracture method. The membrane was submerged into liquid nitrogen and subsequently sliced with a razor blade into a wedge with a wide base and very thin point (<100 μm). 2-part epoxy was used to affix this wedge to a sample pin for imaging. The thin point extended slightly above the pin to isolate it for imaging while maintaining stability during rotation.

In pure absorption mode, the sample was rotated through 1301 projections spanning 180° with 15 s of X-ray exposure at each projection. Detector pixels in LFOV are 64 nm, and camera binning of 2 was used for all measurements to yield reconstructed voxel sizes of 128 × 128 × 128 nm and a subsequent resolution of ∼500 nm. Imaging was performed with monochromatic X-rays at 5.4 keV.

### Pressure-Driven Distillation Testing

Distillation experiments were conducted in a bench-scale dead-end filtration setup with a constant applied hydraulic pressure of 3.4 bar (50 psi), which is below the reported liquid entry pressure of 13.8 bar for the 20 nm PTFE membranes used in this study.^21,36^ All PD experiments were conducted at 22 ± 1 °C. The feed solution was prepared by dissolving NaCl at a concentration of 10 mM in ultrapure water, and the permeate side of the membrane was initially in contact with ultrapure water. Four membrane samples were tested: pristine PTFE, and PTFE coated with 250, 500, and 1000 cycles of TiO_2_. Circular membrane coupons with an active diameter of 1 cm, corresponding to an active area of 0.79 cm^2^, were used for all tests. Feed pressure was supplied using compressed nitrogen gas. The filtration setup consisted of a custom-fabricated polyether ether ketone (PEEK) pressure cell fitted with a membrane support and an O-ring seal to prevent bypass. Permeance was monitored by measuring the change in water volume on the permeate side after 20 h. Membrane salt rejection was assessed by measuring the conductivity in the feed and permeate to verify membrane integrity and identify potential wetting.

### Model Dye Degradation to Probe Catalytic Activity

The photocatalytic performance of the TiO_2_–PTFE membranes with increasing cycles of ALD and a pristine PTFE as a control was evaluated by measuring the decrease in the concentration of methylene blue (MB) using a UV spectrophotometer (Cary UV-4000) following exposure to UV light irradiation (365 nm, 8.0 W). Membranes were cut into 16 mm diameter circles and mounted in Petri dishes with Kapton tape to ensure a flat surface. The membranes were immersed in a solution containing 5 mg/L MB. The lamp was positioned 13 cm above the membrane. Prior to starting the UV lamp, the membranes sat in the dye solution in the dark for 1 h to come to light absorption equilibrium. Then, for an additional hour, the membranes remained submerged in the MB solution under dark conditions to establish their behavior. Absorbance measurements were taken at each of these points with a UV–vis spectrometer. Once UV light exposure commenced, absorbance measurements were taken every 30 min for 2 h. After the initial UV exposures, the samples were left in the dark for 16 h, and their absorbance measurements were taken. Similarly, UV exposure was commenced after the 16 h dark period, and absorbance measurements were taken every 45 min for an additional hour and a half.

### Wettability Tests with Liquid Entry Pressure

Liquid entry pressure measurements were taken to establish the wetting resistance of the membranes following ALD fabrication. The membranes were cut into 13 mm diameter circles and loaded into a stainless steel dead-end membrane cell. The feed side was loaded with a 5 mM NaCl solution and was gently tapped to ensure no bubbles. The cell was attached to a nitrogen gas cylinder, and the pressure was slowly increased to 3.5 bar and maintained there for 30 min. Following this initial pressurization, the pressure was increased every 10–15 min by 0.7 bar. Once water was observed on the permeate end of the cell, the pressure was recorded, and a needle valve was used to release gas from the system.

## Results and Discussion

### Atomic Layer Deposition of TiO_2_ on PTFE Membranes

TiO_2_ was grown on PTFE membranes by using a remote plasma ALD process (Figure 1). Because PTFE consists of a fully fluorinated carbon backbone and is therefore highly chemically inert, remote plasma was employed to introduce reactive oxygen-containing functional groups, such as hydroxyls or carbonyls, onto the surface prior to catalyst deposition.^24–28,31^ Following a brief remote plasma activation step, the reaction chamber was sequentially dosed with titanium tetrachloride (Figure 1, step 1) and water (Figure 1, step 2), with a purge step in between the introduction of each chemical. One complete sequence of precursor dosing and purging constituted a single ALD cycle, and this study performed 250, 500, and 1000 cycles of TiO_2_ ALD on the PTFE membranes.

**Figure 1:**
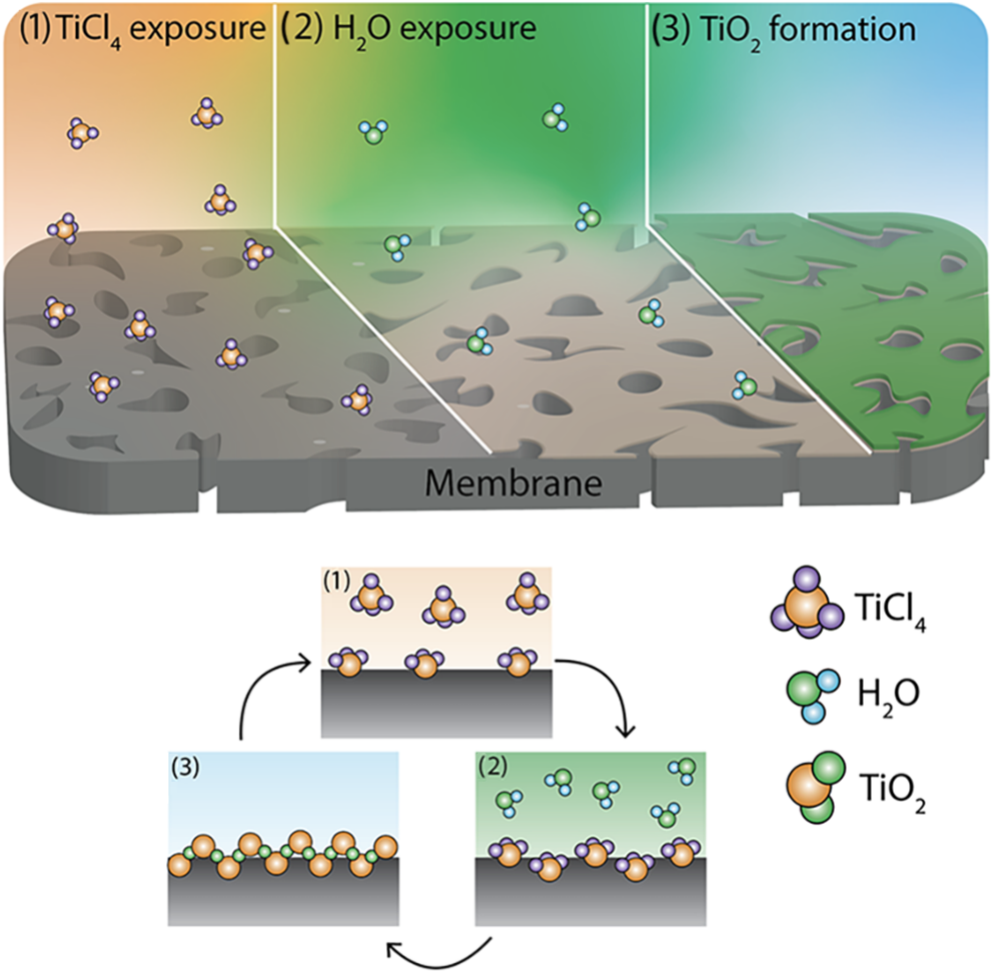
Atomic layer deposition of TiO_2_ on a porous surface: (1) Exposure to titanium tetrachloride, (2) exposure to water vapor, and (3) formation of titanium dioxide on the surface. Repeated cycles are used to deposit layers of a desired thickness.

Following ALD deposition, the top surfaces of the membranes were viewed under SEM to evaluate the morphological changes (Figure 2). Unmodified PTFE membranes exhibit a characteristic fibrous and nodular structure.^24,25,31^ From 250, 500, and 1000 ALD cycles, the apparent PTFE fiber diameters increased by 60, 172, and 189%, respectively, relative to the unmodified fibers (Table S1). The membranes exposed to 1000 ALD cycles also exhibited regions where large clusters of TiO_2_ blocked voids between the PTFE fibers (Figure S1). At higher magnifications, the unmodified membranes displayed fibers with relatively smooth surfaces. In contrast, ALD treatment led to observable particulate growth on PTFE fibers and nodules. Particulates grown at 250 cycles were relatively small and discretely distributed; at 500 cycles, the particulates began to coalesce; and after 1000 cycles, a smooth fiber morphology was observed. Xu et al. observed similar particulate growth on PTFE fibrils following ALD growth of aluminum oxide on PTFE and attributed the observed surface smoothing of the fibers to filling of the spaces between adjacent particles at higher ALD cycles, consistent with our observations.^31^

**Figure 2:**
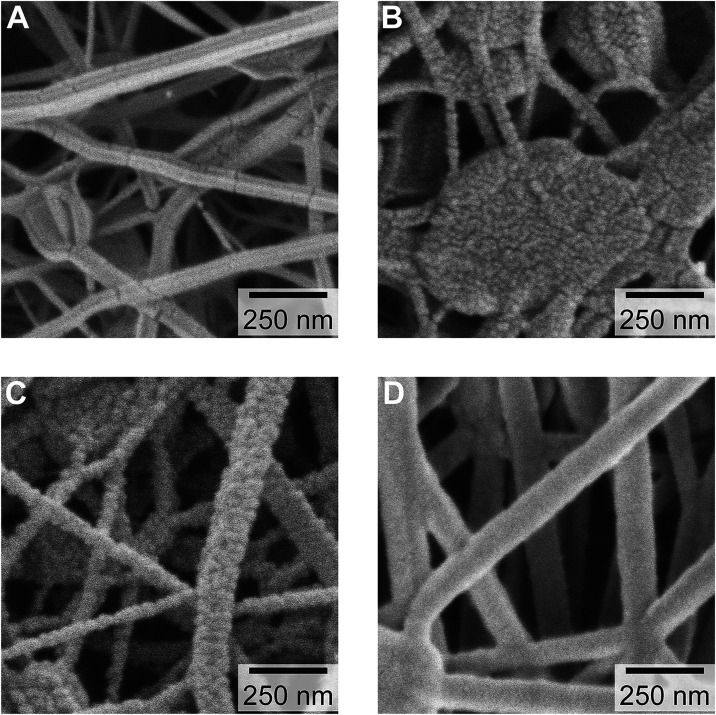
Scanning electron micrographs of pristine PTFE membrane (A) and samples coated with TiO_2_ via ALD with 250 cycles (B), 500 cycles (C), and 1000 cycles (D).

Analysis of the top surface of the membrane via XPS confirmed the chemical composition of the ALD coating. The survey scan (Figure 3A) shows that with increasing ALD cycles, the Ti 2p peak at 458.5 eV systematically increases, while the C 1s peak at 291 eV and the F 1s peak at 688 eV both decrease in intensity. In the carbon core scans (Figure 3B), we see the C–F bond peaks at 290–292 eV disappear as we increase from 250 to 1000 cycles but begin to observe more pronounced peaks for C = O functional groups at 288–290 eV, C–O at 286 eV, and C–C at 284 eV. Similarly to the survey scans, we see the Ti 2p_1/2_ and Ti 2p_3/2_ peaks become more pronounced with increasing cycles (Figure 3C). Since increasing ALD cycles introduce a growing amount of TiO_2_ on the membrane surface, the eventual disappearance of the C–F bond on the surface is consistent with the depth limit of XPS measurements, where only the top 10 nm of the surface can be analyzed.^37^ We assume that the increasing signals for C = O, C–O, and C–C in the core scans are likely from adventitious carbon due to the surface’s exposure to air.^38^ The oxygen core scans (Figure 3D) show two distinct peaks, the first at 529 eV corresponds to lattice oxygen present in TiO_2_,^39^ and the second at 531 eV, which is attributed to surface hydroxyl groups or oxygen bound to adventitious carbon.^40^ Table S2 summarizes survey scan atomic percentages and peak-fitting results from the core scans. Interestingly, even after 1000 cycles, we observed a small fluorine signal in some areas of the analyzed membrane in the XPS survey scan. This may be due to potentially untreated PTFE fibers that lie beneath the coated surface being analyzed during XPS or potential inhomogeneous coverage of the membrane during ALD, which is observed in SEM (Figure S1).

**Figure 3:**
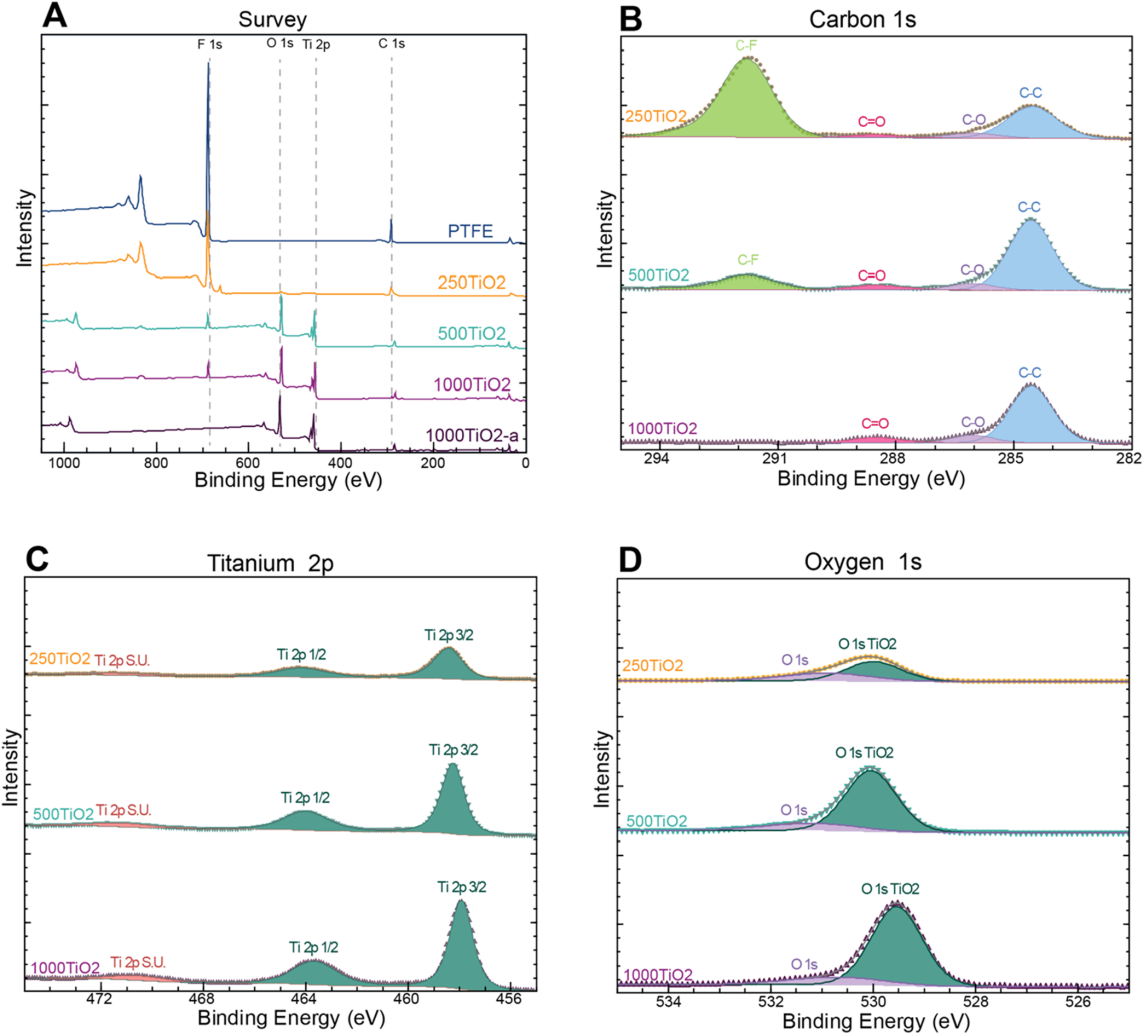
(A) XPS survey spectra of TiO_2_-coated membranes. Core scans and peak fittings of (B) Carbon 1s, (C) Titanium 2p, and (D) Oxygen 1s.

### Penetration Depth of TiO_2_ Coating into PTFE Membranes

Although the successful deposition of TiO_2_ onto the PTFE membrane was confirmed by imaging and spectroscopy, it is important to determine the penetration depth of the precursors into the porous membrane structure. ALD is conventionally thought of as a conformal surface modification technique and has been shown to uniformly coat the entirety of the pores in membrane materials.^34^ However, the selectivity and overall performance of distillation membranes are reliant upon their hydrophobic nature, which allows air to remain trapped in the pore even when the membrane is immersed in liquid water.^20,21^ If the pore is entirely hydrophilic, water will infiltrate the membrane pores, causing nonselective liquid transport. Thus, effective catalytic distillation membranes must limit the penetration of the hydrophilic precursors into the hydrophobic porous membranes. Pathak et al. showed that they were able to control the penetration depth of ALD precursors into porous substrates by limiting dosing times of the precursors.^35^ Following this procedure, precursor dosing times were limited to 0.1 s to control penetration into the PTFE membrane.

To determine the penetration depth of the ALD precursors, cross sections of membranes coated with 1000 cycles of ALD were analyzed with SEM and X-ray energy-dispersive spectroscopy (XEDS) to evaluate the penetration of TiO_2_ coatings. The cross section of the membrane was prepared with a focused ion beam (Figure 4A), and elemental analysis by XEDS showed clear bands of oxygen, titanium, and fluorine (Figure 4B–E). The XEDS mapping indicates that TiO_2_ remained localized near the upper surface of the pores and did not penetrate further into the membrane. To further confirm the localization of the TiO_2_, membranes with 1000 cycles of ALD were imaged with X-ray computed tomography (XCT), which provides submicrometer resolution three-dimensional images of materials’ internal pore network and morphology.^41,42^ Figure 4F shows the reconstructed volume of a sample after 1000 cycles, where the grayscale value is directly proportional to the X-ray absorption. A thin layer of highly absorbing material, likely corresponding to TiO_2_, is observed on the surface of the membrane. Throughout the rest of the PTFE, the average grayscale remains nearly constant, indicating the lack of TiO_2_ penetration. Although deformation during sample preparation prevents precise thickness determination, the surface layer thickness is estimated to be approximately 1 μm (Figure S2). Together, the XCT and XEDS results demonstrate that the TiO_2_ forms a thin surface layer and does not penetrate deeply into the PTFE porous structure.

**Figure 4:**
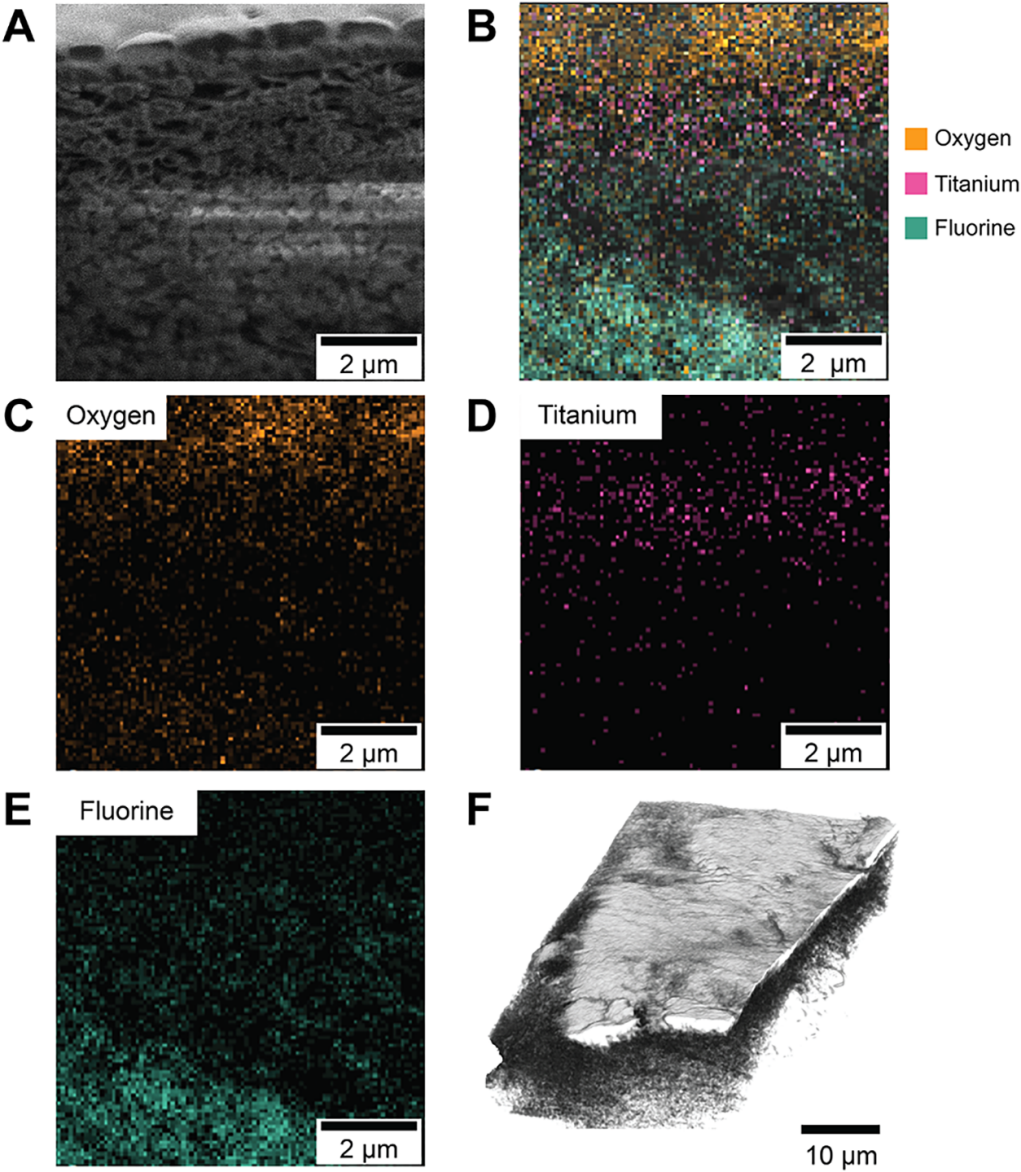
(A) Scanning electron micrograph of PTFE membrane cross sections after 1000 ALD cycles (1000TiO_2_). (B) Corresponding energy-dispersive X-ray spectroscopy elemental map and individual maps for oxygen (C), titanium (D), and fluorine (E). (F) X-ray computed tomography of 1000TiO_2_ membrane, where grayscale intensity is proportional to X-ray attenuation. The bright top surface is associated with a high X-ray absorbing material (TiO_2_), while the black volume is associated with PTFE.

Water contact angle and liquid entry pressure measurements were used to assess changes in membrane wettability following TiO_2_ deposition (Figure 5A). The water contact angle measured at the top surface of the membrane decreased from 147° for unmodified PTFE to 110, 98, and 80° with 250, 500, and 1000 cycles, respectively. Despite this reduction in surface hydrophobicity, the membranes did not readily wick water following modification, suggesting that the underlying air layer within the porous structure remained intact.

**Figure 5:**
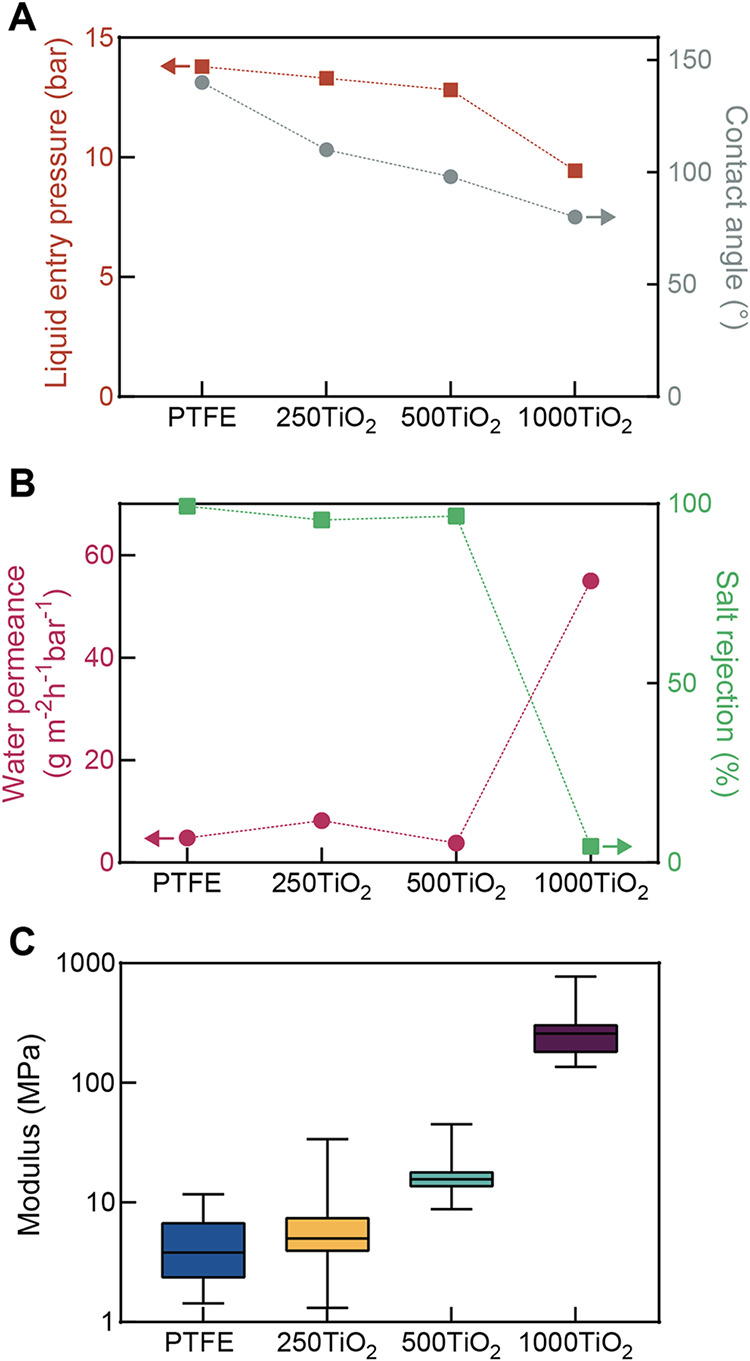
(A) Liquid entry pressure (LEP) and contact angle of the pristine PTFE membrane and membranes coated with increasing cycles of TiO_2_ ALD. (B) Pressure-driven distillation water permeance and salt rejection results of the pristine PTFE membrane and membranes coated with increasing cycles of TiO_2_ ALD. (C) Summary of modulus data of membrane samples taken with nanoindentation. Error bars represent the mean ± 1 s.d. for measurements from at least four areas on the membrane samples.

Liquid entry pressure (LEP) measurements further demonstrated that the TiO_2_ coating did not cause wetting or a loss of distillation functionality. The unmodified PTFE membrane exhibited an LEP of 13.8 bar, while membranes treated with 250, 500, and 1000 ALD cycles showed LEP values of 13.3, 12.8, and 9.4 bar, respectively (Table S1). These results indicate that the trapped air layer inside the membrane is preserved after coating, although increasing coating cycles decreases the LEP. This reduction in the LEP may be caused by changes in hydrophobicity throughout the membrane. Although XCT and SEM-XEDS indicate that TiO_2_ is primarily confined to the membrane surface, subtle changes in wettability within the near-surface pore region may still contribute to changes in the LEP.

We verified that catalytic membranes were capable of desalination using pressure-driven distillation (PD) experiments, where an applied pressure of 3.4 bar was used to drive vapor transport through the membrane.^21,22,36^ The pristine membrane and the membranes modified with 250 and 500 ALD cycles all maintained higher than 95% rejection of 10 mM NaCl, indicating that the ability to separate via distillation was maintained after coating. These membranes also demonstrated permeances around 5.5 g m^–2^ h^–1^ bar^–1^, consistent with prior PD work using these membranes.^36^ In contrast, the membrane treated with 1000 ALD cycles showed a near-complete loss of salt rejection and a 10-fold increase in water permeance, indicating that this membrane experienced liquid breakthrough.

The apparent failure of the membranes treated with 1000 ALD cycles was consistent with SEM measurements taken following LEP measurements, which showed that these membranes exhibited surface cracking (Figure S3), indicating that higher ALD cycles may impact membrane mechanical properties. To probe this behavior further, nanoindentation measurements were performed to determine the elastic moduli of the membranes (Figure 5C). With increasing ALD cycles, the modulus increased markedly from 4.7 MPa for bare PTFE to 310 MPa for the 1000 cycles of TiO_2_ on the PTFE. This indicates that TiO_2_ created a rigid top layer on the membrane, explaining the cracked top surface following LEP measurements. Overall, LEP and distillation performance measurements suggest that the number of cycles should be optimized to enable catalytic behavior while preventing substantial changes to the membrane wetting or mechanical properties.

### Photocatalytic Performance of Fabricated Membranes

TiO_2_ is an effective photocatalyst due to its flat band potential, stability, cost-effectiveness, and low toxicity.^43,44^ When exposed to light, TiO_2_ exhibits photocatalytic activity and produces reactive oxygen species (e.g., hydroxyl or oxygen radicals), which aid in the decomposition of contaminants (Figure 6A).^43,45,46^ The photocatalytic activity of the membranes under UV irradiation was evaluated via degradation of methylene blue (MB), a model contaminant commonly used to benchmark catalytic performance in the literature (Figure 6B).^47–51^ Prior to UV irradiation, the membranes were held under dark conditions for 2 h, with absorbance measurements taken every 2 h. The coated membranes exhibited some slight physical adsorption of the methylene blue, but it was negligible in comparison to the amount removed during UV exposure (Table S3). Additionally, after running 2 h of UV irradiation, the membranes were held in dark conditions for 16 h, during which the absorbance remained unchanged.

**Figure 6:**
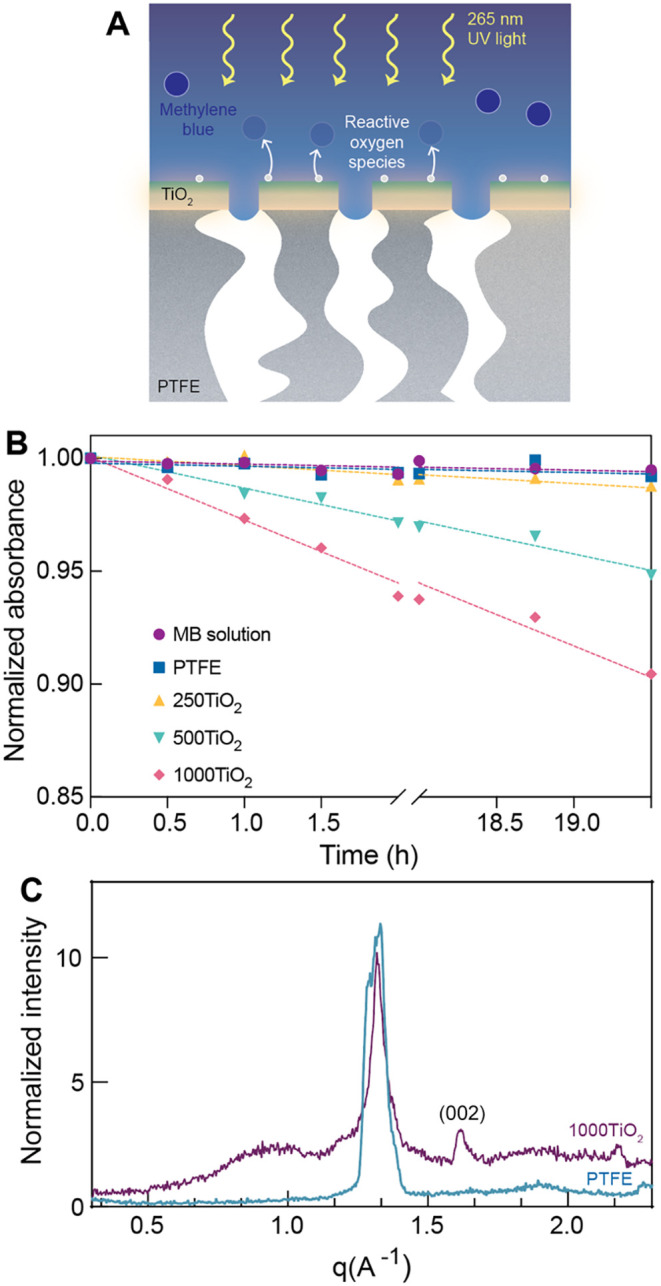
(A) Schematic diagram illustrating photocatalytic degradation of methylene blue in solution with TiO_2_-coated distillation membranes. (B) UV Vis spectroscopy of methylene blue solutions after UV irradiation of TiO_2_ membranes. The initial UV irradiation experiment was 2 h, with absorbance being taken every 30 min. Following the first 2 h, the membranes were kept in the dark for 16 h and then were exposed to UV light for an additional 1.5 h. (C) Grazing-incidence wide-angle X-ray diffraction of pristine PTFE and 1000TiO_2_ PTFE.

For the UV exposure experiments, both the MB control solution and pristine PTFE exhibited negligible degradation (less than 1%), confirming the absence of significant photolysis or adsorption. Increasing the number of ALD cycles on the membranes led to a monotonic increase in the amount of MB degraded under UV irradiation, with 1.2, 5.2, and 9.6% removal of MB observed for membranes coated with 250, 500, and 1000 cycles of TiO_2_, respectively. The degradation rates observed are lower than those of systems highly loaded with photocatalysts but similar to reported values for ultrathin, surface-bound catalytic coatings with limited active material.^52–55^ Zhang et al. reported they saw a 9.6% decrease in methyl orange concentration for ceramic membranes coated with 400 TiO_2_ ALD cycles at a higher temperature of 375 °C, which likely formed more catalytically active anatase species but would have degraded the polyester backing material on our membranes.^52,55^ To facilitate comparison with other studies, the area-normalized degradation rate and TiO_2_ mass loading per area are provided in Table S3.

Photocatalytic reactivity of TiO_2_ is dependent on the crystalline phase, and photocatalytic activity is usually seen in anatase and rutile phases, with anatase exhibiting higher photocatalytic performance.^46,56^ To ensure the formation of anatase TiO_2_, we performed the ALD reactions at 165 °C, which is near the temperature where amorphous TiO_2_ begins to transition to anatase.^46,56^ To probe the crystallinity of the coatings, grazing-incidence X-ray diffraction (GIXRD) measurements were made on the 1000TiO_2_ membrane. The strong reflection in both profiles at 1.3 Å^–1^ arises from crystalline domains of the PTFE membrane (Figure 6C). The profile measured from the 1000TiO_2_ has distinct diffraction peaks arising from the coating, and a broad amorphous peak centered at 0.95 Å^–1^ from the amorphous TiO_2_. The relatively weak reflection at 1.6 Å^–1^ corresponds to a (002) reflection from anatase TiO_2_. The combined amorphous and anatase scattering signatures indicate that the ALD process produces semicrystalline TiO_2_ films at the surface of the PTFE membrane. Polarized optical microscopy images of the coated membranes further confirm the polycrystalline nature of the TiO_2_ films on the membrane surface (Figure S4). This crystallinity was not observed on untreated PTFE. Overall, these results demonstrate that ALD-deposited TiO_2_ imparts photocatalytic activity to otherwise inert PTFE membranes. Even at low deposition temperatures, sufficient anatase formation is achieved for contaminant degradation. However, future optimization may allow for more anatase TiO_2_ formation and higher catalytic degradation rates.

## Conclusions

This work demonstrates a new class of catalytically active distillation membranes enabled by controlled atomic layer deposition on PTFE substrates. By limiting precursor exposure time, TiO_2_ growth was confined to an approximately 1 μm distance from the surface of the membranes, creating a catalytically active interface while preserving the hydrophobic air-trapping pores required for distillation. The fabricated membranes demonstrated pressure tolerances higher than 9 bar without wetting, achieved higher than 95% salt rejection in PD, exhibited chemical signatures consistent with TiO_2_ coatings, and showed catalytic degradation of model dye compounds. These results highlight the ability to integrate catalytic functionality into chemically inert, hydrophobic membranes without compromising their separation performance.

The fabrication strategy presented here provides a platform for the continued development of catalytic distillation membranes that integrate advanced oxidation, high selectivity, and robustness. We show that optimization of catalyst coating parameters (such as the ALD cycle number and precursor pulse times) will be critical for balancing catalytic activity with membrane wettability and mechanical integrity. While this study focused on verifying photocatalytic activity using methylene blue as a standardized benchmark, future work should validate the catalytic removal of representative volatile contaminants and investigate the potential of catalytic coatings for fouling mitigation at the membrane surface. More broadly, future studies should examine performance under realistic desalination and complex wastewater conditions. Continued development of scalable and application-specific coating approaches will be essential for translating catalytic distillation membranes to practical water treatment applications.

## Supplementary Material



## Notes

Membrane fiber diameter, liquid entry pressure, and modulus data (Table S1); XPS peak-fitting results for carbon, titanium, and oxygen core-level spectra and survey scans (Table S2); SEM images showing heterogeneity of the TiO_2_ coating on membrane surfaces (Figure S1); nanoCT cross-sectional image and histogram of oxide layer thickness distribution (Figure S2); SEM images of membranes after liquid entry pressure testing (Figure S3); polarized and optical microscopy images demonstrating TiO_2_ crystallinity (Figure S4); and calculated TiO_2_ mass loading per membrane area and methylene blue degradation rates under dark and UV conditions (Table S3) (PDF)

## References

[1] Karimidastenaei Z., Avellan T., Sadegh M., Klove B., Haghighi A. T. (2022). Unconventional
water resources: Global opportunities and challenges. Sci. Total Environ..

[2] Deshmukh A., Boo C., Karanikola V., Lin S., Straub A. P., Tong T., Warsinger D. M., Elimelech M. (2018). Membrane distillation at the water-energy
nexus: limits, opportunities, and challenges. Energy Environ. Sci..

[3] Shannon M. A., Bohn P. W., Elimelech M., Georgiadis J. G., Marinas B. J., Mayes A. M. (2008). Science and technology for water
purification in the coming decades. Nature.

[4] Xie M., Nghiem L. D., Price W. E., Elimelech M. (2012). Comparison
of the removal of hydrophobic trace organic contaminants by forward
osmosis and reverse osmosis. Water Res..

[5] Basile T., Petrella A., Petrella M., Boghetich G., Petruzzelli V., Colasuonno S., Petruzzelli D. (2011). Review of
Endocrine-Disrupting-Compound Removal Technologies in Water and Wastewater
Treatment Plants: An EU Perspective. Ind. Eng.
Chem. Res..

[6] Elimelech M., Phillip W. A. (2011). The future of seawater
desalination: energy, technology,
and the environment. Science.

[7] Werber J. R., Osuji C. O., Elimelech M. (2016). Materials
for next-generation desalination
and water purification membranes. Nat. Rev.
Mater..

[8] Xie M., Lee J., Nghiem L. D., Elimelech M. (2015). Role of pressure in organic fouling
in forward osmosis and reverse osmosis. J. Membr.
Sci..

[9] Duan Y., Wang R., Shocron A. N., Elimelech M. (2025). Design principles
of catalytic reactive membranes for water treatment. Nat. Water.

[10] Li N., Lu X., He M., Duan X., Yan B., Chen G., Wang S. (2021). Catalytic membrane-based oxidation-filtration
systems for organic
wastewater purification: A review. J. Hazard.
Mater..

[11] Romanos G. E., Athanasekou C. P., Likodimos V., Aloupogiannis P., Falaras P. (2013). Hybrid Ultrafiltration/Photocatalytic Membranes for
Efficient Water Treatment. Ind. Eng. Chem. Res..

[12] Do V. T., Tang C. Y., Reinhard M., Leckie J. O. (2012). Effects of chlorine
exposure conditions on physiochemical properties and performance of
a polyamide membrane--mechanisms and implications. Environ. Sci. Technol..

[13] Do V. T., Tang C. Y., Reinhard M., Leckie J. O. (2012). Degradation of polyamide
nanofiltration and reverse osmosis membranes by hypochlorite. Environ. Sci. Technol..

[14] Nickerson T. R., Antonio E. N., McNally D. P., Toney M. F., Ban C., Straub A. P. (2023). Unlocking the potential of polymeric desalination membranes
by understanding molecular-level interactions and transport mechanisms. Chem. Sci..

[15] Davari S., Omidkhah M., Salari S. (2021). Role of polydopamine in the enhancement
of binding stability of TiO2 nanoparticles on polyethersulfone ultrafiltration
membrane. Colloids Surf., A.

[16] Ning R., Yan Z., Lu Z., Wang Q., Wu Z., Dai W., Fan G., Fu X. (2022). Photocatalytic membrane for in situ enhanced removal
of semi-volatile organic compounds in membrane distillation under
visible light. Sep. Purif. Technol..

[17] Lee S., Shirts M. R., Straub A. P. (2024). Molecular
fingerprint-aided prediction
of organic solute rejection in reverse osmosis and nanofiltration. J. Membr. Sci..

[18] Lim S., Shi J. L., von Gunten U., McCurry D. L. (2022). Ozonation of organic
compounds in water and wastewater: A critical review. Water Res..

[19] Cairney H. T., Hjelvik E. A., Straub A. P. (2024). Impact of Oxidative
Chemicals on
Hydrophobic Porous Membranes Used in Membrane Distillation. ACS Appl. Eng. Mater..

[20] Lopez K. P., Wang R., Hjelvik E. A., Lin S., Straub A. P. (2023). Toward
a universal framework for evaluating transport resistances and driving
forces in membrane-based desalination processes. Sci. Adv..

[21] Nguyen D. T., Lee S., Lopez K. P., Lee J., Straub A. P. (2023). Pressure-driven
distillation using air-trapping membranes for fast and selective water
purification. Sci. Adv..

[22] Lee S., Laris O. A., Hjelvik E. A., Hoek E. M. V., Straub A. P. (2025). High Pressure
Resistance in Omniphobic Distillation Membranes with Re-entrant Nanostructures. Nano Lett..

[23] Madalosso H. B., Machado R., Hotza D., Marangoni C. (2021). Membrane Surface
Modification by Electrospinning, Coating, and Plasma for Membrane
Distillation Applications: A State-of-the-Art Review. Adv. Eng. Mater..

[24] Krumpolec R., Cameron D. C., Homola T., Černák M. (2017). Surface chemistry
and initial growth of Al 2 O 3 on plasma modified PTFE studied by
ALD. Surf. Interfaces.

[25] Lai C.-L., Liou R.-M., Chen S.-H., Huang G.-W., Lee K.-R. (2011). Preparation
and characterization of plasma-modified PTFE membrane and its application
in direct contact membrane distillation. Desalination.

[26] Morra M., Occhiello E., Garbassi F. (1990). Surface characterization of plasma-treated
PTFE. Surf. Interface Anal..

[27] Wilson D. J., Eccles A. J., Steele T. A., Williams R. L., Pond R. C. (2000). Surface
chemistry and wettability of plasma-treated PTFE. Surf. Interface Anal..

[28] Zanini S., Barni R., Pergola R. D., Riccardi C. (2014). Modification of the
PTFE wettability by oxygen plasma treatments: influence of the operating
parameters and investigation of the ageing behaviour. J. Phys. D: Appl. Phys..

[29] Huang R., Liu Z., Woo Y. C., Luo W., Gray S. R., Xie M. (2020). Emerging investigator
series: engineering membrane distillation with nanofabrication: design,
performance and mechanisms. Environ. Sci.: Water
Res. Technol..

[30] Zhang L., Feng Y., Li Y., Jiang Y., Wang S., Xiang J., Zhang J., Cheng P., Tang N. (2022). Stable construction
of superhydrophobic surface on polypropylene membrane via atomic layer
deposition for high salt solution desalination. J. Membr. Sci..

[31] Xu Q., Yang Y., Wang X., Wang Z., Jin W., Huang J., Wang Y. (2012). Atomic layer deposition of alumina
on porous polytetrafluoroethylene membranes for enhanced hydrophilicity
and separation performances. J. Membr. Sci..

[32] Alam J., Alhoshan M., Dass L. A., Shukla A. K., Muthumareeswaran M. R., Hussain M., Aldwayyan A. S. (2016). Atomic layer deposition of TiO2 film
on a polyethersulfone membrane: separation applications. J. Polym. Res..

[33] Chen H., Wu S., Jia X., Xiong S., Wang Y. (2018). Atomic layer deposition
fabricating of ceramic nanofiltration membranes for efficient separation
of dyes from water. AIChE J..

[34] Elam J. W., Xiong G., Han C. Y., Wang H. H., Birrell J. P., Hryn J. N., Pellin M. J., Poco J. F., Satcher J. H. (2006). Atomic
Layer Deposition for the Conformal Coating of Nanoporous Materials. MRS Proc..

[35] Pathak R., Rozyyev V., Shevate R., Mane A. U., Elam J. W. (2023). Controlling
Nanoscale Pore Size and Wall Composition in Polycarbonate Membranes
via Atomic Layer Deposition and Sequential Infiltration Synthesis:
Implications for High Water Permeance. ACS Appl.
Nano Mater..

[36] Nguyen D. T., Lopez K. P., Lee S., Lee J., Hernandez M. T., Straub A. P. (2023). Water Desalination via Pressure-Driven Distillation
with Chlorine-Resistant and Large-Area Polymeric Membranes. Environ. Sci. Technol. Lett..

[37] Greczynski G., Hultman L. (2022). A step-by-step guide
to perform x-ray photoelectron
spectroscopy. J. Appl. Phys..

[38] Grey L. H., Nie H.-Y., Biesinger M. C. (2024). Defining the nature of adventitious
carbon and improving its merit as a charge correction reference for
XPS. Appl. Surf. Sci..

[39] Krishnan P., Liu M., Itty P. A., Liu Z., Rheinheimer V., Zhang M.-H., Monteiro P. J. M., Yu L. E. (2017). Characterization
of photocatalytic TiO2 powder under varied environments using near
ambient pressure X-ray photoelectron spectroscopy. Sci. Rep..

[40] Zhou J.-H., Sui Z.-J., Zhu J., Li P., Chen D., Dai Y.-C., Yuan W.-K. (2007). Characterization
of surface oxygen
complexes on carbon nanofibers by TPD, XPS and FT-IR. Carbon.

[41] Lee S.-H., Chang W.-S., Han S.-M., Kim D.-H., Kim J.-K. (2017). Synchrotron
X-ray nanotomography and three-dimensional nanoscale imaging analysis
of pore structure-function in nanoporous polymeric membranes. J. Membr. Sci..

[42] Scharf J., Chouchane M., Finegan D. P., Lu B., Redquest C., Kim M. C., Yao W., Franco A. A., Gostovic D., Liu Z., Riccio M., Zelenka F., Doux J.-M., Meng Y. S. (2022). Bridging
nano- and microscale X-ray tomography for battery research by leveraging
artificial intelligence. Nat. Nanotechnol..

[43] Nakata K., Fujishima A. (2012). TiO2 photocatalysis:
Design and applications. J. Photochem. Photobiol.,
C.

[44] Xu Z., Wu T., Shi J., Teng K., Wang W., Ma M., Li J., Qian X., Li C., Fan J. (2016). Photocatalytic antifouling
PVDF ultrafiltration membranes based on synergy of graphene oxide
and TiO2 for water treatment. J. Membr. Sci..

[45] Chen S.-L., Wang A.-J., Dai C., Benziger J. B., Liu X.-C. (2014). The effect
of photonic band gap on the photo-catalytic activity of nc-TiO2/SnO2
photonic crystal composite membranes. Chem.
Eng. J..

[46] Chu L., Qin Z., Yang J., Li X. (2015). Anatase TiO2 Nanoparticles with Exposed
{001} Facets for Efficient Dye-Sensitized Solar Cells. Sci. Rep..

[47] Lakshmi S., Renganathan R., Fujita S. (1995). Study on TiO2-mediated photocatalytic
degradation of methylene blue. J. Photochem.
Photobiol., A.

[48] Sangpour P., Hashemi F., Moshfegh A. Z. (2010). Photoenhanced Degradation of Methylene
Blue on Cosputtered M:TiO2 (M = Au, Ag, Cu) Nanocomposite Systems:
A Comparative Study. J. Phys. Chem. C.

[49] Song J., Wang X., Yan J., Yu J., Sun G., Ding B. (2017). Soft Zr-doped TiO(2) Nanofibrous
Membranes with Enhanced Photocatalytic
Activity for Water Purification. Sci. Rep..

[50] Tayade R. J., Natarajan T. S., Bajaj H. C. (2009). Photocatalytic Degradation of Methylene
Blue Dye Using Ultraviolet Light Emitting Diodes. Ind. Eng. Chem. Res..

[51] Tichapondwa S. M., Newman J. P., Kubheka O. (2020). Effect of TiO2 phase on the photocatalytic
degradation of methylene blue dye. Phys. Chem.
Earth.

[52] Lee A., Libera J. A., Waldman R. Z., Ahmed A., Avila J. R., Elam J. W., Darling S. B. (2017). Conformal
Nitrogen-Doped TiO2 Photocatalytic
Coatings for Sunlight-Activated Membranes. Adv.
Sustainable Syst..

[53] Zhang H., Duan Y., Elimelech M., Wang Y. (2025). Scalable catalytic
nanofiltration membranes for advanced water treatment. Nat. Water.

[54] Zhang L., He D., Jiang Y., Li Y., Shi X., Wang S., Xiang J., Zhang J., Cheng P., Tang N. (2022). AgTi nanoparticle
hybrid PVDF membrane with stable and high efficiency antibacterial
performance by atomic layer deposition. Surf.
Interfaces.

[55] Zhang H., Mane A. U., Yang X., Xia Z., Barry E. F., Luo J., Wan Y., Elam J. W., Darling S. B. (2020). Visible-Light-Activated
Photocatalytic Films toward Self-Cleaning Membranes. Adv. Funct. Mater..

[56] Liu G., Wang X., Chen Z., Cheng H. M., Lu G. Q. (2009). The role
of crystal phase in determining photocatalytic activity of nitrogen
doped TiO2. J. Colloid Interface Sci..

