# Supplementary material for: Fabrication of Catalytic Distillation Membranes with Atomic Layer Deposition
Source: ACS Appl Mater Interfaces. 2026 Jul 3;18(27):37835–44. doi: 10.1021/acsami.6c07890 (PMC13383264; doi:10.1021/acsami.6c07890)
Supplement: Supplementary file 1 [file am6c07890_si_001.pdf]

Supporting Information

Fabrication of catalytic distillation membranes with atomic layer deposition

Elizabeth A. Hjelvik[1,2], Kian P. Lopez[3], Samuel D. Marks[2,5], Bryce Knutson[2], Scott C. Minas[4], Madison King[6,7], Joseph C. Roback[3], Matthew Ticknor[2], Ryan C. Hayward[2,3], Michael F. Toney[2,3,8], John Watt[6], Dale L. Huber[1], and Anthony P. Straub[2,9*]

---

[1]Center for Integrated Nanotechnologies, Sandia National Laboratories, Albuquerque, New Mexico 87123, USA

[2]Department of Materials Science and Engineering, University of Colorado Boulder, Boulder, Colorado 80309, USA

[3]Department of Chemical and Biological Engineering, University of Colorado Boulder, Boulder, Colorado 80309, USA

[4]Nanoscience and Microsystems Engineering, University of New Mexico, 210 University Blvd NE, Albuquerque, New Mexico 87131, USA

[5]Advanced Photon Source, Argonne National Laboratory, Lemont, Illinois 60439, USA

[6]Center for Integrated Nanotechnologies, Los Alamos National Laboratory, Los Alamos, New Mexico 87545, USA

[7]Northern Arizona University, Center for Materials Interfaces in Research and Applications, Flagstaff, Arizona 86011, USA

[8]Renewable and Sustainable Energy Institute, University of Colorado Boulder, Boulder, Colorado 80309, USA

[9]Department of Mechanical and Process Engineering, ETH Zürich, Zürich 8092, Switzerland

*Corresponding author, E-mail: astraub@ethz.ch

Table S1. Summary of membrane properties. Fiber diameters were measured from SEM images. Liquid entry pressure (LEP) for each membrane was obtained experimentally. Modulus measurements were obtained from nanoindentation measurements.

Sample	Fiber diameter (nm)	LEP (bar)	Modulus (MPa)
PTFE	66.4±12.5	13.8	4.7±2.7
250TiO2	107±10	13.3±0.3	6.8±6.2
500TiO2	181±23	12.8± 0.3	16.5±7.7
1000TiO2	192±57.3	9.4±0.3	310±180

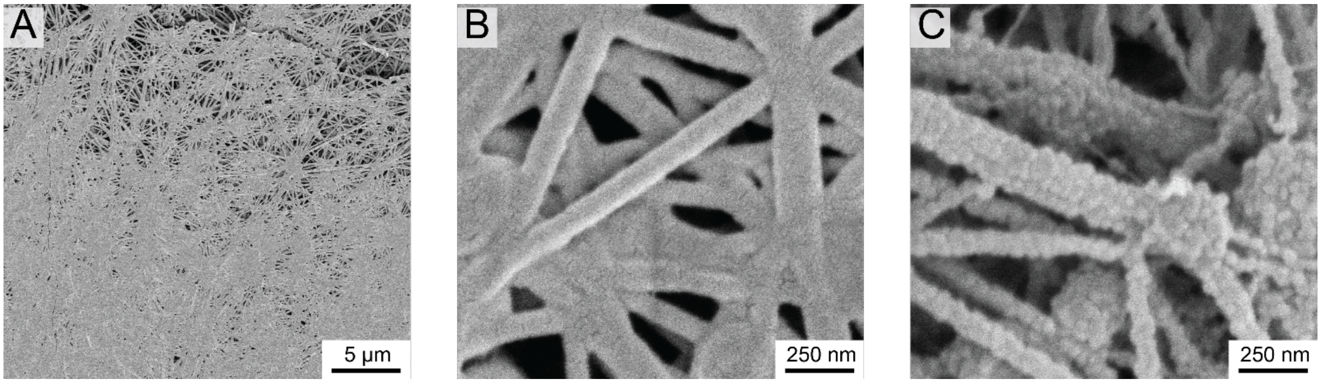

Figure S1. Scanning electron microscopy images of 1000TiO_2 membrane showing heterogeneity in TiO_2 coating after deposition: (A) low magnification; (B,C) high magnification.

Table S2. Summaries of carbon core peak fitting results, titanium core peak fitting results, oxygen core peak fitting results and XPS survey scans. Uncoated PTFE summaries were included for the XPS survey and carbon core scans but not for titanium and oxygen core scans.

	Carbon component %						
**Sample**	**C-C**	**C-O**	**C=O**	**O-C=O**	**C-F**	**C-F$_2$**	**C-F$_3$**
PTFE	1.80	0.87	0.17	1.44	0	89.35	6.36
250TiO$_2$	25.34	4.03	0.01	2.45	0	63.82	4.34
500TiO$_2$	68.70	6.73	1.04	4.43	0.24	17.15	1.70
1000TiO$_2$	80.68	11	1.10	7.19	0.0	0.0	0.02

	Titanium component %			Oxygen component %	
**Sample**	**Ti 2p $_{1/2}$**	**Ti 2p $_{3/2}$**	**Ti 2p S.U.**	**O TiO$_2$**	**O surface**
PTFE	-	-	-	-	-
250TiO$_2$	30.99	62.1	6.91	61.15	38.84
500TiO$_2$	30.87	61.86	7.27	81.8	18.2
1000TiO$_2$	31.05	62.22	6.72	84.12	15.88

	Survey- Atomic %				
**Sample**	**Carbon**	**Fluorine**	**Chlorine**	**Titanium**	**Oxygen**
PTFE	34.85	64.82	0	0	0.36
250TiO$_2$	39.45	55.52	0	0.85	4.16
500TiO$_2$	29.19	10.89	1.21	16.31	42.38
1000TiO$_2$	25.31	1.71	2.34	19.44	51.18

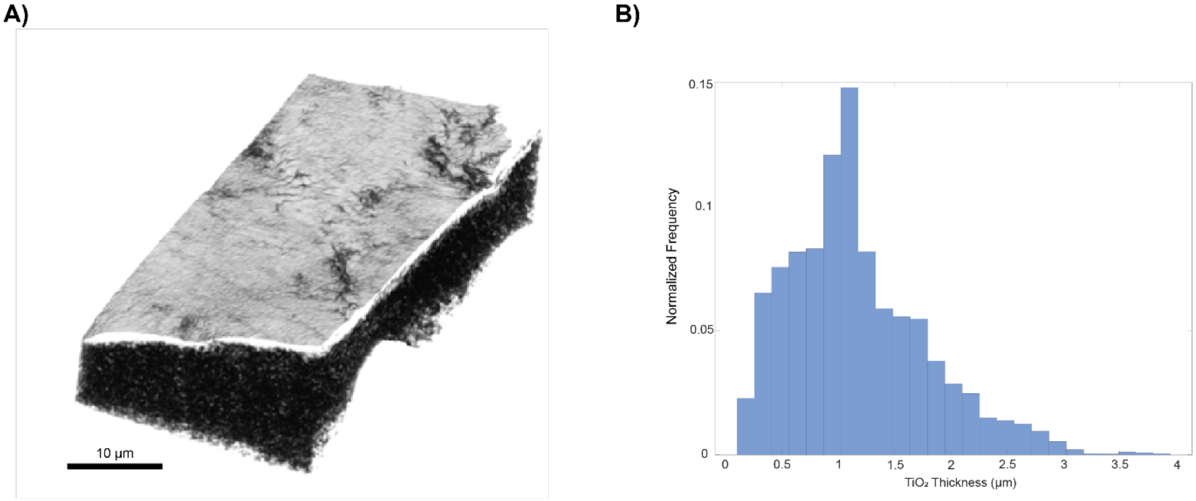

Figure S2. (A) Additional nanoCT image of 1000TiO$_2$ membrane sample. (B) Histogram summarizing oxide layer thickness on membrane. The y-axis is normalized frequency so that the sum of all the bins adds to 1.

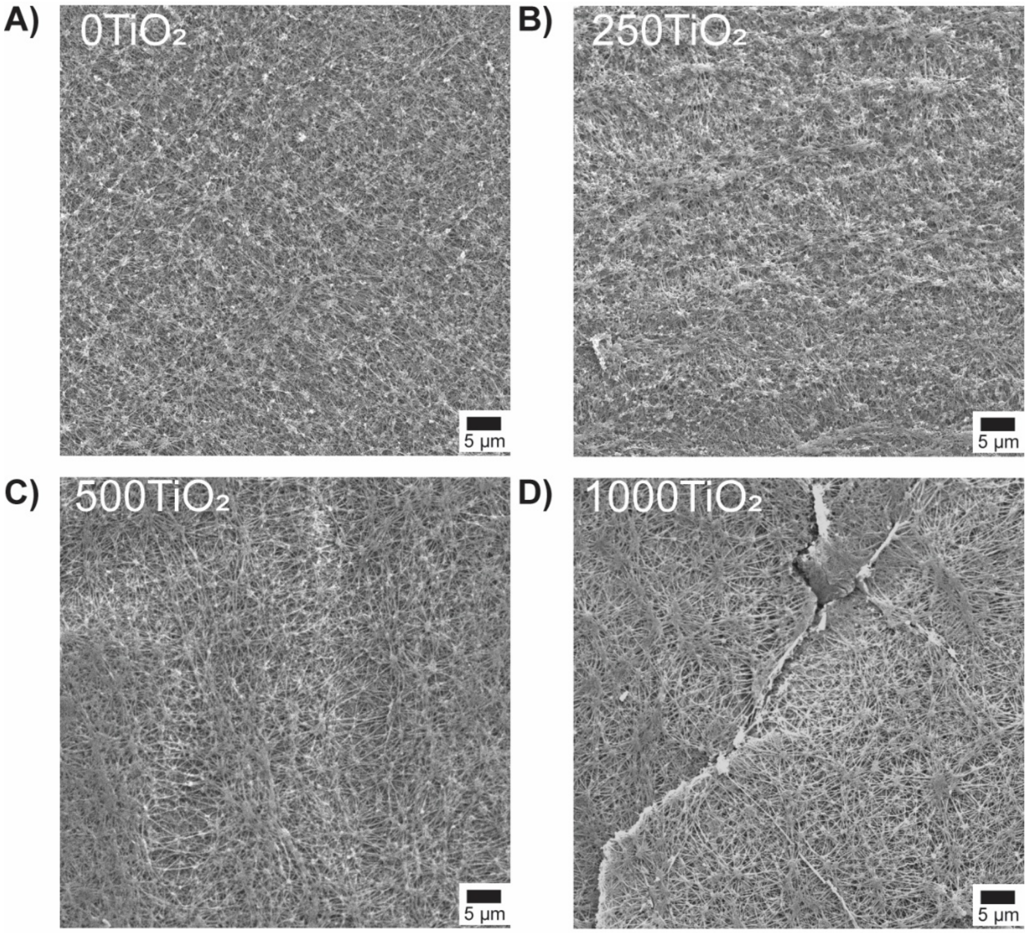

Figure S3. Scanning electron microscopy images of membranes with varying cycles of TiO_2 after liquid entry pressure tests.

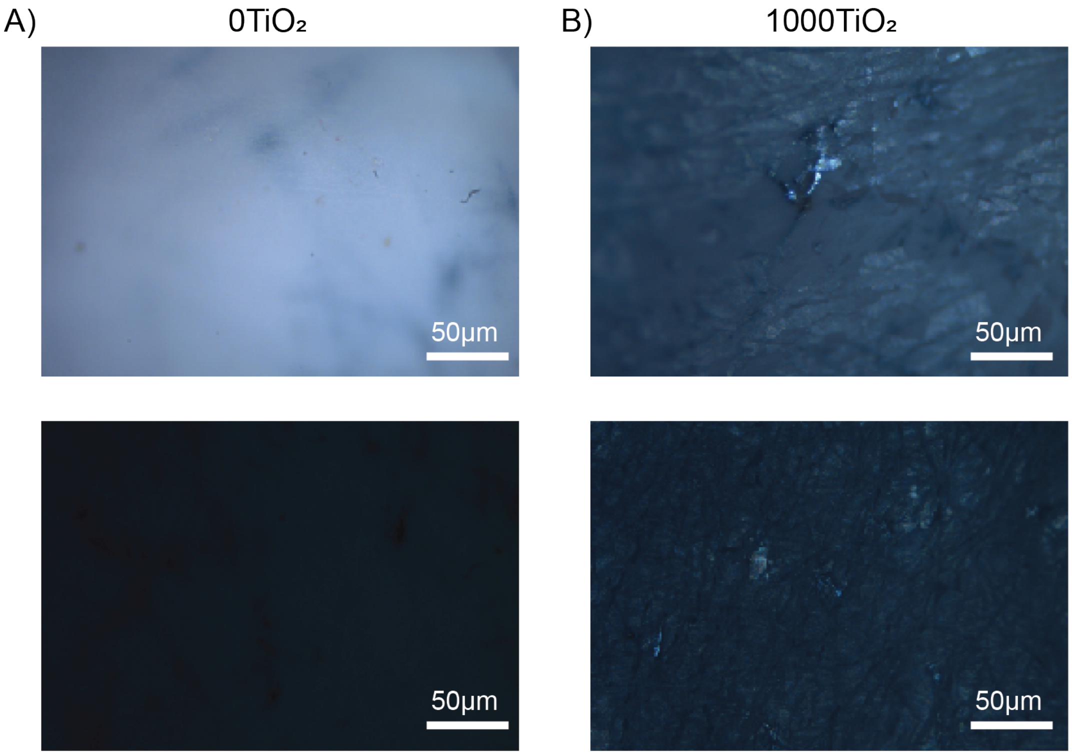

Figure S4. Polarized (bottom) and optical (top) microscopy images of $0TiO_2$ and $1000TiO_2$ membranes at 50x magnification. The lack of birefringence in the $0TiO_2$ samples under polarized microscopy demonstrates no crystal structure. Conversely, the $1000TiO_2$ demonstrates birefringence and crystal formation.

Table S3. Summary of calculated mass of TiO_2 per unit area of membrane, and the calculated removal rate of methylene blue in dark condition and UV exposure conditions. TiO_2 mass per area is calculated assuming 0.05 nm is deposited every cycle assuming a flat surface. Removal is calculated using the slope of experimental degradation curves.

**Sample**	Estimated TiO_2 mass per area ($\mu g\ cm^{-2}$)	Dark removal of MB ($mg\ h^{-1}m^{-2}$)	UV removal of MB ($mg\ h^{-1}m^{-2}$)
$250TiO_2$	3.9	0.57	0.59
$500TiO_2$	7.9	0.64	2.20
$1000TiO_2$	15.6	0.80	4.46
